# Primary Immunodeficiencies: Pathogenetic Advances, Diagnostic and Management Challenges

**DOI:** 10.3390/jcm12144651

**Published:** 2023-07-13

**Authors:** Giorgio Costagliola, Rita Consolini

**Affiliations:** Section of Clinical and Laboratory Immunology, Division of Pediatrics, Department of Clinical and Experimental Medicine, University of Pisa, 56126 Pisa, Italy; giorgio.costagliola@hotmail.com

The field of immunology is rapidly progressing, with new monogenic disorders being discovered every year. The heterogeneity of clinical manifestations and the genetic background of immunodeficiencies brought about the new definition of inborn errors of immunity (IEI), which was adopted by the International Union of Immunological Societies (IUIS) in 2019. This term reflects a considerable change in the viewpoint of immunologists, with a deeper recognition of the non-infectious manifestations of IEI and their atypical presentations. In this current intriguing context, this Special Issue offers an overview of some of the most updated concepts in immunology, ranging from the discussion of some peculiar aspects of well-known entities to the presentation of recently discovered diseases. The Special Issue includes six original research papers and seven review papers submitted from different countries.

As the first contribution to this Special Issue, we provided a review on the autoimmune manifestations of IEI, with a specific focus on the various molecular mechanisms involved in autoimmunity and potential targeted therapeutic strategies [1]. This work analyzes the most common autoimmune manifestations in patients with antibody deficiencies, combined immunodeficiencies, and immune dysregulation disorders, and also introduces some specific monogenic entities that are used as a paradigm of “druggable” IEI.

Following this, other interesting elements can be derived from the review paper by Pieniawska-Śmiech et al., in which some of the most relevant non-infectious presentations of IEI are discussed [2]. Specifically, the authors focus on the role of allergic manifestations, autoimmunity, lymphoproliferation, and malignancies as the first sign of IEI, deeply discussing the role of immune dysregulation.

The atypical presentation of IEI is also the main focus of the paper by Morawska et al., which explores the spectrum of atopic manifestations in patients with selective IgA deficiency (sIgAD). This review gives specific attention to the epidemiological and clinical features of the atopic diseases associated with sIgAD and discusses the most relevant diagnostic aspects [3]. Similarly, selective IgE deficiency is comprehensively discussed in the work by Picado et al. [4], which presents the spectrum of infectious, allergic, autoimmune, and neoplastic manifestations in a large cohort of patients diagnosed with this largely unknown condition.

The other papers in this Special Issue have a major focus on the molecular mechanisms responsible for IEI. Concerning this, the review paper by Romano et al. discusses the role of epigenetic alterations associated with IEI [5]. This paper offers an overview of the epigenetic mechanisms implicated in the regulation of the immune response and the most relevant known epigenetic alterations in IEI.

The review paper by Mertowska et al. [6] deeply discusses the molecular structure and function of the Foxp3 transcription factor and its role in the immune response and in the development of IEI. The work has a specific focus on the pathogenesis of IPEX syndrome, but also presents the intriguing role of FOXP3 in common variable immunodeficiency (CVID).

The paper by Votto et al. [7] deals with the role of gastrointestinal eosinophilic manifestations in patients with IEI. As these manifestations are still underdiagnosed, the article offers an interesting view on when to suspect gastrointestinal eosinophilic manifestations in individuals diagnosed with IEI, as well as when to suspect an IEI in those presenting with isolated gastrointestinal eosinophilic involvement.

Another paper analyzing a rare and poorly recognized entity is the cohort study by Alberio et al. [8], in which the clinical and laboratory features of patients with the DiGeorge-like clinical phenotype in the absence of the classical 22q11.2 deletion are described. This study evidences some new copy number variants associated with the Di George-like phenotype, strongly suggesting the use of array CGH in patients presenting with this phenotype to better identify the genotype-to-phenotype correlations.

Finally, the wide spectrum of antibody deficiencies is the main focus of four research papers and a review paper. The study by Więsik-Szewczyk et al. [9] analyzes a cohort of adult patients with CVID associated with autoimmunity or isolated infectious manifestations, reporting some peculiarities in the immunophenotype of those affected by autoimmune diseases. Indeed, the authors describe a tendency for lymphopenia, reduced NK cells, and low levels of regulatory T cells and Th17 cells in patients with autoimmunity, thus contributing to the elucidation of the immunological heterogeneity of the disease. Two papers published in this Special Issue were by the research group of Diaz Alberola et al. [10,11]. In the first paper [10], the epidemiologic, clinical, and immunological features of CVID-associated giardiasis are reviewed, evidencing that patients with giardiasis more commonly have reduced IgA levels and lower levels of switched memory B cells. The second paper published by this group [11] is original research describing a patient with CVID associated with a de novo IKZF1 variant, in which a reduced humoral response against the SARS-CoV-2 vaccine was demonstrated in the presence of an adequate T-cell response against the pathogen. Another study investigating the relationship between IEI and SARS-CoV-2 is the original research by Pieniawska-Śmiech et al. [12]. In this study, the incidence of COVID-19, its clinical course, and the anti-SARS-CoV-2 serologic response in a cohort of patients with IEI are analyzed, demonstrating a low rate of severe infections in the study cohort.

Finally, the paper by Sgrulletti et al. [13] focuses on the clinical evolution of pediatric patients with unclassified primary antibody deficiencies, highlighting the need for an appropriate follow-up to promptly identify those who will progress to definite IEI.

To conclude, the present Special Issue represents an overview of the current immunological scenario, and deals with different innovative concepts and clinical and research approaches. Indeed, the expanding availability of immunological and genetic testing offers the opportunity to identify new disease entities and elucidate the function of new genes involved in the development and regulation of the immune response. In this continuously evolving field, both researchers and clinicians need to be constantly updated on the most relevant innovations, and with this Special Issue we hope to have contributed to this extremely relevant topic.
